# Sedentary Behavior, Physical Activity, and Hypertension: Genetic Liability and the Role of Metabolic Factors

**DOI:** 10.1002/clc.70380

**Published:** 2026-06-15

**Authors:** Ye Cao, Jia Wang, Nan Xia, Qing Hao, Yi Feng

**Affiliations:** ^1^ Renmin Hospital Hubei University of Medicine Shiyan Hubei P. R. China; ^2^ Hubei Clinical Medical Research Center for Atherosclerotic Cardiovascular Diseases Hubei University of Medicine Shiyan Hubei Province P. R. China; ^3^ Department of Pharmacy, Renmin Hospital Hubei University of Medicine Shiyan Hubei P. R. China; ^4^ Department of Pulmonary and Critical Care Medicine, Renmin Hospital Hubei University of Medicine Shiyan Hubei P. R. China

**Keywords:** hypertension, mediation analysis, Mendelian randomization, physical activity, sedentary behavior

## Abstract

**Background:**

Whether sedentary behavior and physical activity causally influence hypertension remains uncertain. We used Mendelian randomization (MR) to examine whether genetically predicted domain‐specific sedentary behaviors and leisure‐time physical activity are associated with hypertension risk and to explore potential cardiometabolic pathways.

**Methods:**

We performed a two‐sample MR using genome‐wide significant, LD‐independent SNPs as instruments for domain‐specific sedentary traits and physical activity. Hypertension was defined by the FinnGen R9 endpoint I9_HYPTENSESS. Pleiotropy‐robust estimators, outlier procedures and two‐step MR mediation analyses were applied.

**Results:**

Genetically proxied leisure screen time (LST) was associated with a higher risk of hypertension (IVW OR = 1.181, 95% CI 1.076–1.296, *p* = 4.70e^−4^). Two‐step MR suggested that body mass index (BMI) and type 2 diabetes (T2D) may partly account for this association, with estimated mediated proportions of 54.7% and 14.7%, respectively, although these estimates should be interpreted cautiously. No robust associations were observed for moderate‐to‐vigorous physical activity (MVPA) or sedentary behavior at work (SDW). The MVPA estimate was directionally protective, but these null findings remained inconclusive given lower instrument informativeness.

**Conclusion:**

Genetically proxied LST was associated with higher hypertension risk and may represent a plausible, potentially actionable prevention target. BMI and T2D may partly account for this association. No robust associations were observed for MVPA or SDW, but these null findings should not be interpreted as definitive evidence of no effect. Further validation in prospective, multi‐ancestry, and mechanistic studies is needed.

## Graphical abstract

Leisure screen time showed the most consistent genetic association with hypertension, with possible partial mediation by body mass index and type 2 diabetes.

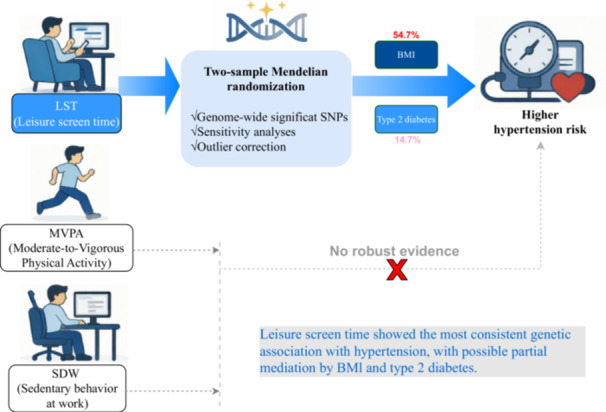

## 1 Introduction

Hypertension is a global epidemic and a major risk factor for cardiovascular disease, imposing substantial burdens on healthcare systems and society [1, 2]. Even a modest 5‐mmHg reduction in systolic blood pressure is associated with an approximately 10% lower relative risk of major cardiovascular events [3]. Despite the widespread availability of antihypertensive therapies, population‐level control rates remain suboptimal, likely reflecting the persistent influence of modifiable lifestyle factors [4]. Epidemiologic evidence implicates high sodium intake, smoking, unhealthy diet, and insufficient physical activity in the development of hypertension [5], underscoring the need for prevention strategies that target behavior.

Physical activity comprises any skeletal‐muscle movement that expends energy, whereas sedentary behavior is defined as waking activity with energy expenditure ≤ 1.5 metabolic equivalents (METs) in a sitting, reclining, or lying posture. Meta‐analyses of observational studies suggest that lower physical activity and higher sedentary time are associated with increased hypertension risk [6, 7, 8, 9]. However, these associations may be biased by confounding and reverse causation, leaving causality unresolved.

Physical activity and sedentary behavior are closely linked with obesity, type 2 diabetes (T2D), and hyperlipidemia [10, 11, 12]—established risk factors for hypertension. These metabolic traits may mediate part of any behavior–hypertension relationship, yet the extent and pathways of mediation remain unclear. Clarifying these mechanisms could inform more effective prevention and treatment strategies.

Mendelian randomization (MR) uses genetic variants as instrumental variables (IVs) to strengthen causal inference between exposures and outcomes [13]. Because genetic variants are allocated at conception, MR can mitigate confounding and reverse causation, offering more robust estimates of causal effects than conventional observational designs.

Accordingly, we conducted a two‐sample MR analysis to evaluate whether genetically predicted domain‐specific sedentary behaviors and leisure‐time physical activity are associated with hypertension. Because hypertension is a major modifiable determinant of cardiovascular morbidity and mortality, clarifying the causal relevance of specific behavioral phenotypes may help refine prevention targets and cardiometabolic risk stratification in clinical cardiovascular care. Exposure and outcome Genome‐wide association study (GWAS) datasets were harmonized, and the inverse variance weighted (IVW) method was used as the primary estimator, and complementary sensitivity analyses included MR‐Egger, weighted median, weighted mode, MR‐PRESSO, and RadialMR. Multiple testing was controlled across exposures, and pleiotropy and heterogeneity were evaluated for each trait. Two‐step MR was further applied to assess potential mediation by body mass index (BMI), T2D, and hyperlipidemia.

## 2 Materials and Methods

### 2.1 Study Design

This study followed the STROBE‐MR guidelines [14]; the completed checklist is provided in Table S7. It was designed as a human genetic causal inference analysis rather than a mechanistic experimental investigation. The aim was not to provide direct biological validation, but to address whether these behavioral traits show evidence consistent with a causal relationship with hypertension at the population level. MR inference relies on three assumptions: (i) the genetic instruments are strongly associated with the exposure; (ii) the instruments are independent of confounders of the exposure–outcome relationship; and (iii) the instruments affect the outcome only through the exposure.

**Figure 1:**
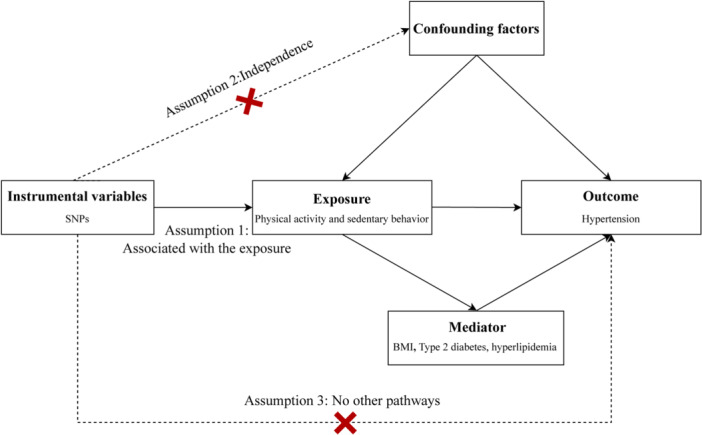
The flowchart of this MR study.

As depicted in Figure 1, we conducted a two‐sample MR to evaluate the causal effects of domain‐specific sedentary behaviors and physical activity on hypertension. We then applied two‐step MR to assess whether BMI, T2D, and hyperlipidemia mediate observed associations.

### 2.2 Data Sources

Summary‐level instruments for sedentary domains and physical activity were drawn from a genome‐wide meta‐analysis of 51 cohorts using self‐reported domain‐specific questionnaires, covering leisure screen time (LST), sedentary behavior at work (SDW), sedentary commuting (SDC), and leisure‐time moderate‐to‐vigorous physical activity (MVPA) [15]. LST denotes nonoccupational screen‐based leisure time (e.g., television viewing, video gaming, and recreational computer use); SDW reflects workplace sedentary time; SDC reflects sedentary time during commuting; and MVPA captures leisure‐time activity at moderate to vigorous intensity.

The hypertension outcome was the FinnGen R9 endpoint I9_HYPTENSESS, defined from the national registry and ICD‐based records [16]. Summary GWAS for potential mediators—BMI, T2D, and hyperlipidemia—were obtained from the IEU OpenGWAS repository. Details of all outcome and mediator datasets are provided in Table 1.

**Table 1:** Basic information on the GWAS datasets for the outcome and mediators.

Trait	GWAS‐ID	Year	Population	Number of SNPs	Sample size
Hypertension	finn‐b‐I9_HYPTENSESS	2021	European	16 380 443	ncase: 42 857 ncontrol: 162 837
Body mass index	ieu‐b‐40	2018	European	2 336 260	681 275
Type 2 diabetes	ebi‐a‐GCST006867	2018	European	5 030 727	ncase: 61 714 ncontrol: 1178
Hyperlipidemia	ukb‐b‐17462	2018	European	9 851 867	ncase: 3439 ncontrol: 459 571

### 2.3 Selection of IVs

We employed three criteria to identify independent SNPs associated with exposures. First, SNPs reaching a genome‐wide significance threshold of *p* < 5 × 10^−8^ were selected. Second, we used pairwise linkage disequilibrium (LD) analysis to assess independence among chosen SNPs. SNPs located within 1000 kb and exhibiting LD (*r* ^2^ > 0.001) were excluded. Third, we used the *F*‐statistic to assess the strength of each SNP. SNPs with *F*‐statistics greater than 10 were considered sufficiently robust to mitigate potential biases [17]. Furthermore, the LDtrait tool was used to identify phenotype associations suggestive of potential confounding or pleiotropy [18]. For the final selected instruments, SNP‐level *F*‐statistics and *R* ^2^ were derived from the SNP–exposure associations, and exposure‐level mean *F*‐statistics and cumulative *R* ^2^ were summarized for transparency.

### 2.4 Data Analysis

We employed four different methodologies for MR analysis: IVW, MR‐Egger, weighted median, and weighted mode. IVW, widely recognized for its widespread application and effectiveness, was chosen as the primary analytical approach in this study [19]. Its high statistical power relies on the assumption of the validity of all SNPs as IVs. Associations with a *p* < 0.05 were considered suggestive of significance, and associations with a *p* < 0.017 (Bonferroni correction, *p* = 0.05/3) were deemed statistically significant [20].

Separate two‐sample MR analyses were performed to examine the causal relationships between significant exposure factors and metabolic factors, as well as between metabolic factors and hypertension. To assess the mediating role of metabolic factors in the causal relationship between significant exposure factors and hypertension, the causal effect of these factors on hypertension, termed the total effect, was denoted as β1. The causal effect of significant exposure factors on metabolic factors was denoted as β2, while the effect of metabolic factors on hypertension was denoted as β3. Here, β2 × β3 represents the mediation effect, and β2 × β3/β1 indicates the proportion of this mediating effect.

We conducted sensitivity analyses to assess the robustness of our findings. MR‐Egger regression was employed to detect horizontal pleiotropy, and Cochran's *Q‐*test was used to evaluate heterogeneity [21, 22]. Radial MR and MR‐PRESSO methods were used to detect outliers if heterogeneity or pleiotropy existed [23, 24]. After removing outliers, the IVW method was employed to estimate causal effects, with simultaneous reassessment of heterogeneity and pleiotropy. Finally, a leave‐one‐out sensitivity analysis was performed to examine the impact of individual SNPs on the overall estimates.

## 3 Results

### 3.1 Selection of IVs

Applying the above criteria, we identified 115, 16, and 9 SNPs for LST, MVPA, and SDW, respectively; no genome‐wide significant SNPs were available for SDC (Table S1), which was therefore not analyzed further. After LDtrait screening, six LST SNPs and one MVPA SNP were excluded. Following harmonization, 95 LST SNPs entered the primary MR analysis, and RadialMR subsequently identified 14 additional LST outliers, yielding 81 SNPs in the corrected analysis (Table S2). The final instrument sets used in the main analyses, therefore, comprised 81 SNPs for LST, 13 for MVPA, and 8 for SDW. Exposure‐level mean F‐statistics were 130.04, 141.08, and 127.56, with cumulative *R* ^2^ values of 0.0215, 0.00361, and 0.00320, respectively. SNP‐level *F*‐statistics and *R* ^2^ values for the final selected instruments are provided in Table S3, and exposure‐level summaries are reported in Table S4 to facilitate transparent assessment of instrument validity and strength.

### 3.2 MR Results of Sedentary Behavior and Physical Activity on Hypertension

**Figure 2:**
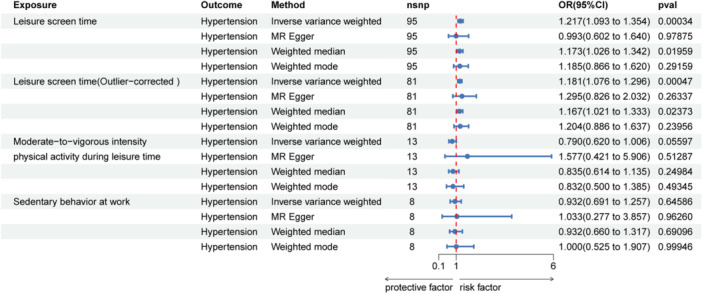
Causal relationships between sedentary behavior, physical activity, and hypertension.

**Figure 3:**
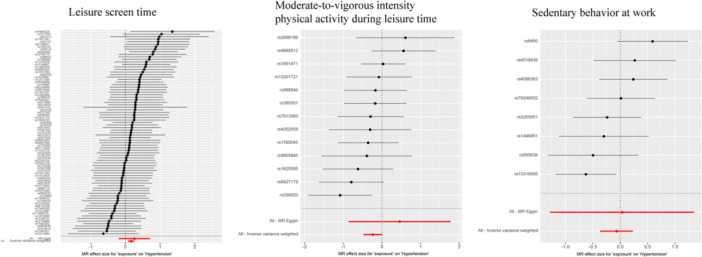
Forest plots of the relationships of sedentary behavior and physical activity with hypertension.

Through two‐sample MR analysis, we found that genetically predicted LST showed an association consistent with a potential causal effect on hypertension (IVW: OR = 1.217, 95% CI: 1.093–1.354, *p* = 3.37e^−4^) (Figures 2, 3, S1, Tables 2, S5). The other three MR methods showed the same direction of effect. By contrast, we did not find robust evidence supporting associations of genetically predicted MVPA (IVW: OR = 0.790, 95% CI 0.620–1.006, *p* = 0.0560) or SDW (IVW: OR = 0.932, 95% CI: 0.691–1.257, *p* = 0.646) with hypertension (Figures 2, 3, S1, Tables 2, S5), although the MVPA point estimate remained directionally protective. Given the smaller instrument sets and substantially lower cumulative *R* ^2^ for MVPA and SDW than for LST, these null findings should be interpreted as inconclusive rather than as evidence of no causal effect.

**Table 2:** The main results of the study.

Exposure	Number of SNPs	IVW method		MR‐Egger regression		MR‐presso		Cochran's Q test *p* value
		OR (95% CI)	*p* value	Egger intercept	*p* value	RSSobs	Global test *p* value	
Leisure screen time	95	1.217 (1.093, 1.354)	3.37E‐04	0.00553	0.419	150.62	3.33E‐04	3.41E‐04
Leisure screen time (Outlier‐corrected)	81	1.181 (1.076, 1.296)	4.70E‐04	−0.00250	0.681	68.75	0.862	0.830
Moderate‐to‐vigorous intensity physical activity during leisure time	13	0.790 (0.620, 1.006)	0.0560	−0.0174	0.319	15.17	0.389	0.388
Sedentary behavior at work	8	0.932 (0.691, 1.257)	0.646	−0.00334	0.880	15.51	0.125	0.079

A sensitivity analysis was then conducted. Although horizontal pleiotropy for LST was not detected by the MR‐Egger intercept test (*p* = 0.419), significant heterogeneity was discovered by Cochran's *Q*‐test (*p* = 3.41e^−4^) (Tables 2, S5). Consequently, the MR‐PRESSO method was utilized to attempt outlier identification for heterogeneity elimination. However, the global test achieved statistical significance without identifying significant outliers (*p* = 3.33e^−4^). Subsequently, the Radial MR method was employed again for outlier detection, revealing 14 outliers (Table S2). After their exclusion, the LST‐hypertension association persisted (IVW: OR = 1.181, 95% CI: 1.076–1.296, *p* = 4.70e^−4^) (Figures 2, 3, S1, Tables 2, S5). Additionally, neither heterogeneity nor horizontal pleiotropy was detected by Cochran's *Q*‐test, MR‐Egger intercept test, nor MR‐PRESSO. Finally, leave‐one‐out analysis showed the robustness of this causal relationship (Figure S2). For MVPA and SDW, sensitivity analysis did not reveal significant heterogeneity or horizontal pleiotropy, and leave‐one‐out analysis also demonstrated the robustness of the results (Figures 3, S1, S2, Tables 2, S5). However, given the smaller instrument sets and lower cumulative *R* ^2^ for these traits, the corresponding estimates should still be interpreted cautiously.

### 3.3 MR Results of Two‐Step MR Analysis

In the first step of the mediation analysis, genetically predicted LST was positively associated with BMI (IVW: OR = 1.160, 95% CI: 1.105–1.218, *p* = 2.49 e^−9^), T2D (IVW: OR = 1.217, 95% CI: 1.093–1.354, *p* = 3.35 e^−4^), and hyperlipidemia (IVW: OR = 1.004, 95% CI: 1.002–1.006, *p* = 6.90 e^−6^) (Table S6). Although some heterogeneity was observed, no horizontal pleiotropy was detected, confirming the robustness of the results.

When hypertension was considered as the outcome, positive associations were observed for BMI (IVW: OR = 1.845, 95% CI: 1.707–1.995, *p* = 7.44e^−54^), and T2D (IVW: OR = 1.132, 95% CI: 1.086–1.180, *p* = 5.91e^−9^) (Table S6). Although some heterogeneity was present, no evidence of horizontal pleiotropy was detected. For hyperlipidemia, only two SNPs were available, and both had *F*‐statistics < 10. Therefore, this mediator was not carried forward because the resulting MR estimates were considered insufficiently reliable.

Two‐step MR suggested that the association between LST and hypertension may be partly accounted for by BMI and T2D. Specifically, the estimated mediated effects were 0.091 and 0.024, corresponding to mediated proportions of 54.7% and 14.7%, respectively (Table 3). These mediation estimates should be interpreted cautiously because two‐step MR relies on additional assumptions, may be sensitive to pleiotropy and instrument limitations, does not provide a precise mechanistic partitioning of the total effect, and does not establish definitive biological sequencing.

**Table 3:** Results of the two‐step MR mediation analysis.

Exposure	Mediation	Outcome	β1 (95%CI)	β2 (95%CI)	β3 (95%CI)	Mediating effect (95%CI)	Mediating ratio (%)
Leisure screen time	Body mass index	Hypertension	0.166 (0.073,0.259)	0.148 (0.100,0.197)	0.613 (0.535,0.690)	0.091 (0.059, 0.123)	54.7
	Type 2 diabetes	Hypertension	0.166 (0.073,0.259)	0.196 (0.089,0.303)	0.124 (0.082,0.166)	0.024 (0.009, 0.040)	14.7

*Note:* β1, total effect; β2, the causal effect of leisure screen time on metabolic factors; β3, the causal effect of metabolic factors on hypertension; Mediating effect: β2 × β3.

## 4 Discussion

This study evaluated whether genetically predicted domain‐specific sedentary behaviors and leisure‐time physical activity are associated with hypertension risk using a two‐sample MR framework. Among the evaluated exposures, LST showed the only robust association consistent with a potential causal effect on hypertension after pleiotropy‐robust sensitivity analyses and outlier correction. By contrast, evidence for MVPA and SDW was not robust, and SDC lacked genome‐wide significant instruments for analysis. Two‐step MR further suggested that BMI and T2D may partly account for the LST‐hypertension association. These findings should be interpreted as human genetic evidence supporting biological plausibility at the population level, rather than as mechanistic proof.

Our findings are broadly consistent with prior observational evidence linking sedentary behavior to elevated blood pressure. A 2015 meta‐analysis of 31 high‐quality studies reported that, despite 18 studies (58.1%) showing no significant association individually, the pooled estimates indicated that each additional hour/day of sedentary time was associated with + 0.06 mmHg systolic and + 0.20 mmHg diastolic blood pressure [9]. Extending this, a 2018 meta‐analysis of 58 studies (*n* = 1 071 967) showed a linear increase in hypertension risk with greater sedentary time: + 4% risk per hour/day of sedentary time and + 6% per hour/day of television viewing [8]. In line with these domain‐specific observations, our genetic analysis identified a robust association for LST but not for other sedentary domains.

By contrast, we did not find robust evidence supporting an inverse association of leisure‐time MVPA with hypertension, as suggested in previous observational syntheses [25, 26, 27]. The MVPA point estimate was directionally protective, but mean *F*‐statistics above 10 do not fully resolve concerns about instrument informativeness. Compared with LST, MVPA, and SDW were represented by fewer SNPs and markedly lower cumulative *R* ^2^, which, together with likely measurement error in questionnaire‐based phenotypes, may have reduced power and attenuated estimates toward the null. The same concern applies to SDW. Accordingly, these null findings are better interpreted as inconclusive rather than definitively negative, because although the conventional F‐statistic threshold was exceeded, the MVPA and SDW instruments remained relatively weak in terms of explained variance; larger device‐based GWAS with stronger and more informative instruments will therefore be needed to clarify any causal effects.

Two‐step MR suggested that BMI and T2D may partly account for the association between LST and hypertension, providing biologically plausible support for two linked cardiometabolic pathways [28, 29]. Greater screen‐based sedentary behavior may plausibly promote adiposity and dysglycemia, which in turn may contribute to elevated blood pressure through sympathetic activation, RAAS activation, sodium retention, endothelial dysfunction, and low‐grade inflammation [30, 31, 32, 33, 34, 35, 36, 37]. However, the mediated proportions should be regarded as approximate and hypothesis‐generating. Two‐step MR is sensitive to pleiotropy, weak instruments, and model misspecification, and MR can support biological plausibility but cannot determine the temporal or molecular sequencing of these pathways. Similar caution applies to hyperlipidemia: because only two weak instruments were available, we could not evaluate this mediator reliably, but lipid‐related pathways remain biologically plausible and warrant investigation in future studies with stronger instruments or multivariable MR.

This study has several advantages. Leveraging a two‐sample MR design reduces confounding and reverse causation, strengthening causal inference for the relationships between sedentary behavior, physical activity, and hypertension. Exposures and outcome were drawn from largely independent GWAS sources, limiting sample overlap, and instrument strength and validity were scrutinized with a comprehensive suite of robustness checks. Beyond total effects, two‐step MR enabled an exploration of potentially relevant cardiometabolic pathways linking LST to hypertension.

Several limitations merit consideration. First, behavioral phenotypes were derived from questionnaire‐based meta‐analytic GWAS across multiple cohorts, so phenotype heterogeneity and measurement error are likely and may have attenuated some effect estimates. Second, although the mean *F*‐statistics for the final MVPA and SDW instruments exceeded conventional thresholds, these traits were represented by fewer SNPs and markedly lower cumulative *R* ^2^ than LST, which may have reduced statistical power and biased estimates toward the null. Third, the mediation framework should be interpreted cautiously because two‐step MR relies on additional assumptions. Fourth, because hyperlipidemia could not be reliably evaluated with the available instruments, the present mediation analysis should be regarded as partial rather than exhaustive. Fifth, although exposure and outcome datasets were largely independent, residual sample overlap cannot be fully excluded. Finally, the predominance of European ancestry in the source GWAS limits generalizability to other populations. Future studies should prioritize device‐based phenotypes, stronger instruments for MVPA and occupational sedentary behavior, and explicit validation in ancestrally diverse datasets, including multi‐ancestry GWAS consortia and large population biobanks, to test transportability and improve precision. Summary‐level data also precluded evaluation of nonlinear dose‐response relationships, subgroup effects, and individual‐level personalization.

Overall, these findings provide human genetic evidence that LST is relevant to hypertension risk and suggest that adiposity and dysglycemia may partly account for this relationship. From a clinical standpoint, the main implication lies less in immediate genetic risk prediction than in prioritizing an actionable exposure for cardiometabolic prevention. In practice, LST could be incorporated into hypertension prevention and cardiovascular risk‐management programs as a brief lifestyle metric alongside diet, physical activity, adiposity, and sleep. A pragmatic implementation strategy would be to include a brief screening of daily LST in hypertension clinics or digital lifestyle programs, followed by counseling on reducing screen exposure, replacing prolonged sitting with intermittent light activity, and addressing concurrent weight and glycemic management. At present, the genetic liability estimates reported here are not ready for individual‐level personalization; rather, they help identify LST as a plausible population‐level prevention target that can inform behavioral counseling and future precision‐prevention research. These implications should be viewed as complementary to, rather than a substitute for, established blood pressure management strategies.

## 5 Conclusion

In this two‐sample MR analysis, genetically proxied LST showed an association consistent with a potential causal effect on hypertension, with BMI and T2D emerging as possible partial mediators. No robust associations were detected for MVPA or SDW, but these null findings should be interpreted cautiously given the lower informativeness of their instruments. Overall, these findings are consistent with a potential causal role of LST in hypertension and highlight it as a plausible, potentially actionable target for prevention, while warranting validation in multi‐ancestry, device‐based, and mechanistic studies.

## Author Contributions

Design, data analysis, and manuscript writing: Ye Cao and Jia Wang. Reviewing and visualization: Nan Xia. Supervision, reviewing, and editing: Qing Hao and Yi Feng. All authors contributed to the article and approved the submitted version.

## Funding

The authors have nothing to report.

## Ethics Statement

This study did not require ethical approval as publicly available data were analyzed.

## Conflicts of Interest

The authors declare no conflicts of interest.

## Supporting information


**Figure S1:** Scatter plots of the relationships of sedentary behavior and physical activity with hypertension.

**Figure S2:** The leave‐one‐out sensitivity analysis of sedentary behavior and physical activity with hypertension.

**Table S1:** Initial genome‐wide significant instrumental variables for each exposure.

**Table S2:** Excluded instrumental variables and reasons for exclusion.

**Table S3:** Final instrumental variables used in the primary MR analyses, with SNP‐level F‐statistics and R2.

**Table S4:** Exposure‐level summary of instrument strength for the final MR instruments.

**Table S5:** The results of MR analysis.

**Table S6:** The results of mediation MR analysis.

**Table S7:** The STROBE‐MR checklist of this study.

## Data Availability

The data that support the findings of this study are openly available in the Open GWAS project and FinnGen study at https://gwas.mrcieu.ac.uk and https://www.finngen.fi.
