# Supplementary figures and images for: Sedentary Behavior, Physical Activity, and Hypertension: Genetic Liability and the Role of Metabolic Factors
Source: Clin Cardiol. 2026 Jun 15;49(6):e70380. doi: 10.1002/clc.70380 (PMC13267988; doi:10.1002/clc.70380)

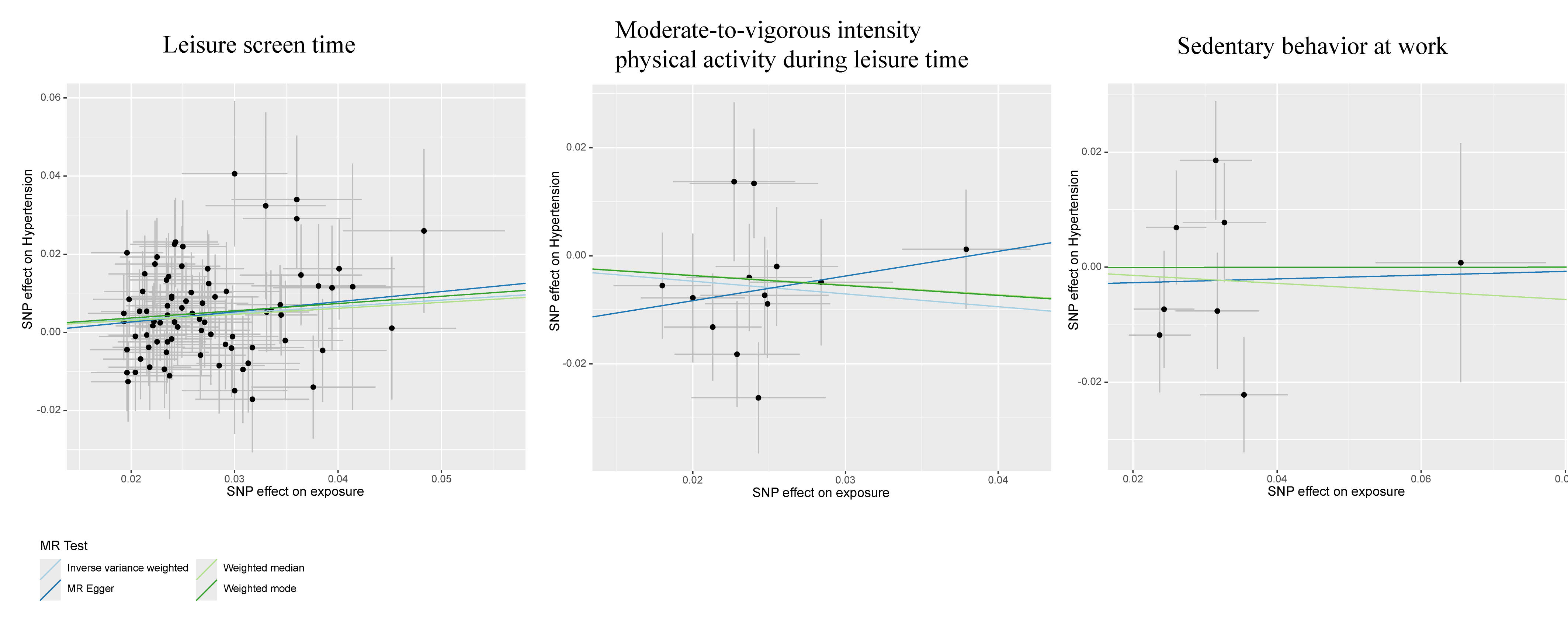

Supplement: Supplementary file 1 — Figure S1: Scatter plots of the relationships of sedentary behavior and physical activity with hypertension. [file CLC-49-e70380-s005.tif]

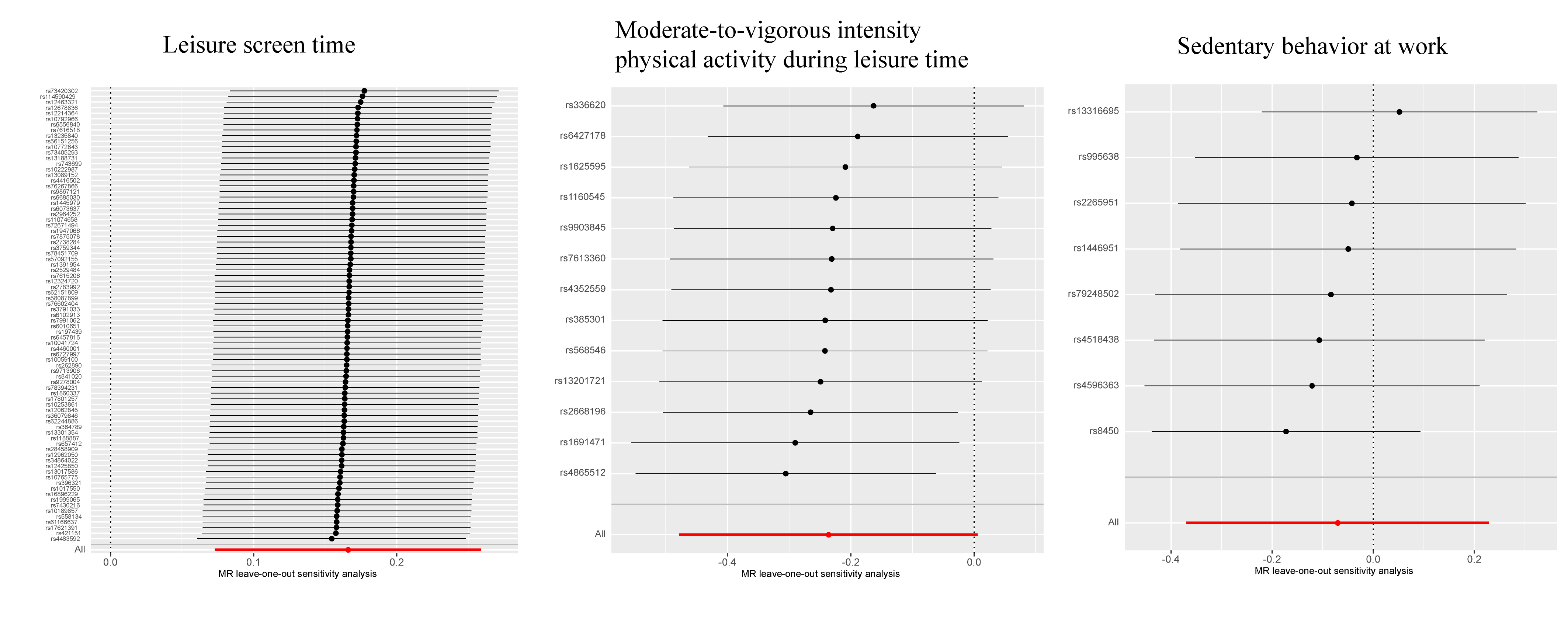

Supplement: Supplementary file 2 — Figure S2: The leave‐one‐out sensitivity analysis of sedentary behavior and physical activity with hypertension. [file CLC-49-e70380-s002.tif]
