# Supplementary material for: Sedentary Behavior, Physical Activity, and Hypertension: Genetic Liability and the Role of Metabolic Factors
Source: Clin Cardiol. 2026 Jun 15;49(6):e70380. doi: 10.1002/clc.70380 (PMC13267988; doi:10.1002/clc.70380)
Supplement: Supplementary file 9 — Table S7: The STROBE‐MR checklist of this study. [file CLC-49-e70380-s009.docx]

| **Table S7 STROBE-MR checklist of recommended items to address in reports of Mendelian randomization studies** | | | | |
| --- | --- | --- | --- | --- |
|  | | | | |
| **Item No.** | **Section** | **Checklist item** |  | **Relevant text from manuscript** |
| 1 | TITLE and ABSTRACT | Indicate Mendelian randomization (MR) as the study’s design in the title and/or the abstract if that is a main purpose of the study |  | The term "Mendelian randomization" was included in the abstract. |
| **INTRODUCTION** | | | | |
| 2 | Background | Explain the scientific background and rationale for the reported study. Is causality between exposure and outcome plausible? Justify why MR is a helpful method to address the study question. |  | Introduction, Paragraph 1-3 |
| 3 | Objectives | State specific objectives clearly, including pre-specified causal hypotheses (if any). State that MR is a method that, under specific assumptions, intends to estimate causal effects |  | Introduction, Paragraph 4-5 |
| **METHODS** | | | | |
| 4 | Study design and data sources | Present key elements of the study design early in the article. Consider including a table listing sources of data for all phases of the study. For each data source contributing to the analysis, describe the following: |  |  |
|  | a) | Setting: Describe the study design and the underlying population, if possible. Describe the setting, locations, and relevant dates, including periods of recruitment, exposure, follow-up, and data collection, when available. |  | Materials and Methods, Section "Study design" and "Data sources". |
|  | b) | Participants: Give the eligibility criteria, and the sources and methods of selection of participants. Report the sample size, and whether any power or sample size calculations were carried out prior to the main analysis |  | Information about the GWAS studies was provided in the Materials and Methods, Sections "Study design" and "Data sources". |
|  | c) | Describe measurement, quality control and selection of genetic variants |  | Materials and Methods, Section "Selection of instrumental variables". |
|  | d) | For each exposure, outcome, and other relevant variables, describe methods of assessment and diagnostic criteria for diseases |  | Materials and Methods, Section "Data sources". |
|  | e) | Provide details of ethics committee approval and participant informed consent, if relevant |  | Not applicable to our study. |
| 5 | Assumptions | Explicitly state the three core IV assumptions for the main analysis (relevance, independence and exclusion restriction) as well assumptions for any additional or sensitivity analysis |  | Materials and Methods, Section "Study design" and "Data analysis". |
| 6 | Statistical methods: main analysis | Describe statistical methods and statistics used |  |  |
|  | a) | Describe how quantitative variables were handled in the analyses (i.e., scale, units, model) |  | Materials and Methods, Sections "Study design" and "Data analysis". |
|  | b) | Describe how genetic variants were handled in the analyses and, if applicable, how their weights were selected |  | Materials and Methods, Section "Study design" and "Data analysis". |
|  | c) | Describe the MR estimator (e.g. two-stage least squares, Wald ratio) and related statistics. Detail the included covariates and, in case of two-sample MR, whether the same covariate set was used for adjustment in the two samples |  | Materials and Methods, Section "Data analysis". |
|  | d) | Explain how missing data were addressed |  | Not applicable to our study |
|  | e) | If applicable, indicate how multiple testing was addressed |  | Materials and Methods, Section "Data analysis". |
| 7 | Assessment of assumptions | Describe any methods or prior knowledge used to assess the assumptions or justify their validity |  | Materials and Methods, Section " Study design". |
| 8 | Sensitivity analyses and additional analyses | Describe any sensitivity analyses or additional analyses performed (e.g. comparison of effect estimates from different approaches, independent replication, bias analytic techniques, validation of instruments, simulations) |  | Materials and Methods, Section "Data analysis ". |
| 9 | Software and pre registration |  |  |  |
|  | a) | Name statistical software and package(s), including version and settings used |  | Materials and Methods, Section "Data analysis". |
|  | b) | State whether the study protocol and details were pre-registered (as well as when and where) |  | Not applicable to our study. |
| **RESULTS** | | | | |
| 10 | Descriptive data |  |  |  |
|  | a) | Report the numbers of individuals at each stage of included studies and reasons for exclusion. Consider use of a flow diagram |  | Materials and Methods, Sections "Study design" and "Data sources". |
|  | b) | Report summary statistics for phenotypic exposure(s), outcome(s), and other relevant variables (e.g. means, SDs, proportions) |  | Materials and Methods, Sections "Study design" and "Data sources". |
|  | c) | If the data sources include meta-analyses of previous studies, provide the assessments of heterogeneity across these studies |  | Not applicable to our study |
|  | d) | For two-sample MR: |  |  |
|  |  | i. Provide justification of the similarity of the genetic variant-exposure associations between the exposure and outcome samples |  | Not applicable to our study |
|  |  | ii. Provide information on the number of individuals who overlap between the exposure and outcome studies |  | Not applicable to our study |
| 11 | Main results |  |  |  |
|  | a) | Report the associations between genetic variant and exposure, and between genetic variant and outcome, preferably on an interpretable scale |  | Results, Sections "MR results of sedentary behavior and physical activity on hypertension", "MR results of two-step MR analysis", Table 2-3, Table S5-6 and Figure 2-3. |
|  | b) | Report MR estimates of the relationship between exposure and outcome, and the measures of uncertainty from the MR analysis, on an interpretable scale, such as odds ratio or relative risk per SD difference |  | Results, Sections "MR results of sedentary behavior and physical activity on hypertension", "MR results of two-step MR analysis", Table 2-3, Table S5-6 and Figure 2-3. |
| 12 | Assessment of assumptions |  |  |  |
|  | a) | Report the assessment of the validity of the assumptions |  | Table 2 and Table S5-6. |
|  | b) | Report any additional statistics (e.g., assessments of heterogeneity across genetic variants, such as I2, Q statistic or E-value) |  | Results, Sections "MR results of sedentary behavior and physical activity on hypertension", "MR results of two-step MR analysis", Table 2, and Table S5-6. |
| 13 | Sensitivity analyses and additional analyses |  |  |  |
|  | a) | Use sensitivity analyses to assess the robustness of the main results to violations of the assumptions |  | Results, Sections "MR results of sedentary behavior and physical activity on hypertension", "MR results of two-step MR analysis", Table 2, Table S5-6 and Figure S1-2. |
|  | b) | Report results from other sensitivity analyses (e.g., replication study with different dataset, analyses of subgroups, validation of instrument(s), simulations, etc) |  |  |
|  | c) | Report any assessment of direction of causality (e.g., bidirectional MR) |  |  |
|  | d) | When relevant, report and compare with estimates from non-MR analyses |  |  |
|  | e) | Consider any additional plots to visualize results (e.g., leave-one-out analyses) |  |  |
| **DISCUSSION** | | | | |
| 14 | Key results | Summarize key results with reference to study objectives |  | Discussion, Paragraph 1 |
| 15 |  | Discuss limitations of the study, taking into account the validity of the MR assumptions, other sources of potential bias, and imprecision. Discuss both direction and magnitude of any potential bias, and any efforts to address them |  | Discussion, Paragraph 7 |
| 16 | Interpretation |  |  |  |
|  | a) | a) Give a cautious overall interpretation of results considering objectives and limitations Compare with results from other relevant studies. |  | Discussion, Paragraph 2-3 |
|  | b) | b) Discuss underlying biological mechanisms that could be modelled by using the genetic variants to assess the relationship between the exposure and the outcome. |  | Discussion, Paragraph 4-5 |
|  | c) | c) Discuss whether the results have clinical or policy relevance, and whether interventions could have the same size effect |  |  |
| 17 | Generalizability | Discuss the generalizability of the study results (a) to other populations, (b) across other exposure periods/timings, and (c) across other levels of exposure |  | Discussion, Paragraph 7 |
| **OTHER INFORMATION** | | | | |
| 18 | Funding | Describe sources of funding and the role of funders in the present study and, if applicable, sources of funding for the databases and original study or studies on which the present study is based |  | Section “Funding”. |
| 19 | Data and data sharing | Provide the data used to perform all analyses or report where and how the data can be accessed, and reference these sources in the article. Provide the statistical code needed to reproduce the results in the article, or report whether the code is publicly accessible and if so, where |  | Section "Data Availability Statement". |
| 20 | Conflicts of Interest | All authors should declare all potential conflicts of interest |  | Section “Competing interests” |
